# Organic–Inorganic Hybrid Materials: Tailoring Carbon Dioxide-Based Polycarbonate with POSS-SH Crosslinking

**DOI:** 10.3390/polym16070983

**Published:** 2024-04-04

**Authors:** Yue Li, Jianyu Liu, Rui Qu, Hongyi Suo, Miao Sun, Yusheng Qin

**Affiliations:** 1 College of Chemistry and Chemical Engineering, Yantai University, Yantai 264005, China; lyyantai@s.ytu.edu.cn (Y.L.); liujianyu@ytu.edu.cn (J.L.);; 2 Institute of Materials, Yantai University, Yantai 264005, China

**Keywords:** carbon dioxide, polycarbonate, poss-sh, thiol-ene click reaction, hybrid materials

## Abstract

A novel functional polycarbonate (PAGC), characterized by the presence of double bonds within its side chain, was successfully synthesized through a ternary copolymerization of propylene oxide (PO), allyl glycidyl ether (AGE), and carbon dioxide (CO_2_). Polyhedral oligomeric silsesquioxanes octamercaptopropyl (POSS-SH) was employed as a crosslinking agent, contributing to the formation of organic–inorganic hybrid materials. This incorporation was facilitated through thiol-ene click reactions, enabling effective interactions between the POSS molecules and the double bonds in the side chains of the polycarbonate. Scanning electron microscopy (SEM) and energy-dispersive X-ray spectroscopy (EDS) confirmed a homogeneous distribution of silicon (Si) and sulfur (S) in the polycarbonate matrix. The thiol-ene click reaction between POSS-SH and the polycarbonate led to a micro-crosslinked structure. This enhancement significantly increased the tensile strength of the polycarbonate to 42 MPa, a notable improvement over traditional poly (propylene carbonate) (PPC). Moreover, the cross-linked structure exhibited enhanced solvent resistance, expanding the potential applications of these polycarbonates in various plastic materials.

## 1. Introduction

CO_2_-based polycarbonate is widely used in packaging bags and agricultural mulching films [1,2] because of its advantages such as biodegradation, biocompatibility, and adjustable performance [3,4,5,6]. Additionally, its environmentally friendly production process holds significant industrial promise. However, the range of epoxides currently used for copolymerization with carbon dioxide is quite limited, with the primary focus being on a few epoxides like propylene oxide (PO) and cyclohexane oxide (CHO) [7,8,9]. Polymers derived from this route frequently exhibit a deficiency in functional groups that would permit subsequent chemical modifications, thus constraining their application potential [10,11]. Therefore, there is an imperative demand to develop carbon dioxide-based polycarbonates with reactive functional groups to improve their adaptability and performance.

Currently, the predominant strategies for the functional modification of carbon dioxide-based polycarbonates involve the copolymerization of carbon dioxide with epoxide monomers bearing specific functional groups or the employment of post-polymerization modification techniques [12,13,14,15,16,17,18]. The latter strategy is particularly distinguished and is widely used as a method to modify the properties of polymers. This approach includes a range of processes, such as click reactions, amidation, etherification, amination, and esterification [19,20,21]. Alagi et al. introduced a novel method for synthesizing graft copolymers [22]. They converted the vinyl double bond of the PVCHC side chain into a carboxylic acid group, which was partially neutralized with tetrabutyl ammonium hydroxide to obtain active carboxylic acid sites. These sites were subsequently grafted with PPC to form PVCHC-g-PPC and PMAA-g-PPC brush copolymers for further modification. Alagi et al. also described a type of CO_2_-based thermoplastic elastomer (CO_2_-TPE) using a sequential synthesis strategy [17]. Unlike traditional thermoplastic elastomers, the double bonds on allyl glycidyl ether (AGE) monomers serve as reaction sites for post-functionalization of the polymers. By introducing light-responsive groups and a small amount of diborate (DABE) as a dynamic cross-linking agent, the resulting CO_2_-TPEs exhibit robust self-healing properties at room temperature. These new post-polymerization modification strategies have enriched the structure and properties of carbon dioxide-based polymers, and also provided more possibilities for expanding their applications.

In this study, we prepared a CO_2_/PO/AGE ternary copolymer by introducing AGE as the third monomer. Traditional PPC lacks active reactive groups, leading to difficulties in improving its mechanical and thermal properties through post-modification strategies in practical applications. Introducing double bond reaction sites serves as a foundational step for the subsequent incorporation of functional groups, thereby significantly boosting the material’s potential for property enhancement.

The incorporation of organic and inorganic components in hybrid materials aims to enhance material properties through various interactions, either chemical (such as covalent, ionic, and coordination bonds) or physical (like hydrogen bonds). This approach holds significant importance in the realm of polymer modification [23,24,25,26]. Polyhedral oligomeric silsesquioxane (POSS) represents a novel class of organic–inorganic hybrid materials [26,27,28]. POSS readily engages in reactions with the organic phase, forming tightly bonded chemical connections that enhance its compatibility with organic matrices [29]. Its distinctive structure, a cage-like regular octahedron, exhibits robust stability and affords flexibility in structural design. The introduction of rigid POSS into a polymer matrix can induce alterations in polymer chain mobility and morphology [30,31,32,33,34]. The introduction of POSS-SH initiates a chemical reaction with the double bond present in the polymer side chain, leading to the formation of a cross-linking structure. This cross-linking is further reinforced through physical mechanisms facilitated by a photo initiator under UV irradiation, resulting in the establishment of a more stable three-dimensional network structure. As the degree of cross-linking increases, the mechanical strength and thermal properties of the materials are correspondingly enhanced. In this study, reactive double bond sites were introduced into polycarbonate, and POSS-SH was incorporated to investigate the subsequent changes in the mechanical and thermal properties of the polycarbonate materials. The findings provide valuable insights for the further application of carbon dioxide-based polycarbonates.

## 2. Experimental Section

### 2.1. Materials

Ethanol, dichloromethane (DCM), and HPLC tetrahydrofuran (THF) were purchased from Energy Chemical, Shanghai, China. Another batch of THF (analytical reagent grade) was acquired from Tianjin Fuyu Fine Chemical Co., Ltd., Tianjin, China. Methanol and hydrochloric acid were obtained from Yantai Sanhe Chemical., Yantai, China. (3-Mercaptopropyl) trimethoxysilane (MPT) and benzoin dimethyl ether (DMPA) were purchased from Macklin Chemical., Shanghai, China. All the above reagents were used without further purification. Propylene oxide (PO) (Energy Chemical., Shanghai, China) and allyl glycidyl ether (AGE) (Meryer Chemical., Shanghai, China) were refluxed with calcium hydride (CaH_2_) (Macklin Chemical., Shanghai, China) for 24 h and subsequently distilled. Carbon dioxide (99.999% purity) was supplied by Feiyuan Special Gas (Yantai, China) and used as received. Zinc glutarate (ZnGA) was synthesized according to previously published procedures [35].

### 2.2. Measurements

Nuclear magnetic resonance spectroscopy (^1^H-NMR) was performed using a JEOL 400 YH spectrometer (JEOL, Tokyo, Japan) operating at 400 MHz with deuterated chloroform (CDCl_3_) serving as the solvent. Fourier transform infrared spectroscopy (FTIR) measurements were conducted on a Shimadzu IRAffinity-1S spectrometer (Shimadazu, Kyoto, Japan) using the liquid film technique. Molecular weight and molecular weight distributions were determined by gel permeation chromatography (GPC) on a Waters 2414 system (Waters, MA, USA), employing tetrahydrofuran as the eluent at 35 °C with a flow rate of 1 mL/min, calibrated against polystyrene standards.

The tensile properties, including tensile strength and elongation at break (Eb), were measured using a 410R250 Tension Instrument (Suns, Shenzhen, China). The analysis was carried out at ambient temperature (20 °C) with a crosshead speed of 5 mm/min on dry films cut into dumbbell-shaped specimens measuring 12 mm × 2 mm × 0.4 mm.

Differential scanning calorimetry (DSC) was conducted on a Netzsch DSC-200 F3 instrument (Netzsch, Selb, Germany), with a ramp rate of 10 °C/min over a temperature range from −50 °C to 150 °C under a nitrogen atmosphere. Thermogravimetric analysis (TGA) was performed using a Netzsch TG 209 F3 (Netzsch, Selb, Germany), with a heating rate of 10 °C/min from 30 °C to 600 °C, also under a nitrogen atmosphere. Dynamic mechanical thermal analysis (DMTA) was executed on a TA Instruments Q800 (TA Instrument, New Castle, DE, USA), at a frequency of 1 Hz, and a heating rate of 5 °C/min within the temperature range of −30 °C to 150 °C.

Scanning electron microscopy (SEM) investigations of the fracture surfaces were carried out using a JEOL JSM-7900F microscope (JEOL, Tokyo, Japan) at an accelerating voltage of 5 kV. The surfaces were prepared by fracturing the samples under liquid nitrogen to preserve the morphology. Tapping mode atomic force microscopy (TM-AFM) was performed on a NTEGRA Prima instrument (NT-MDT, Moscow, Russia), with amplitude settings ranging from 5 to 25 and a scanning frequency of 1.01 Hz.

### 2.3. Synthesis of Carbon Dioxide-Based Polycarbonate Using PO and AGE

**Scheme 1:**
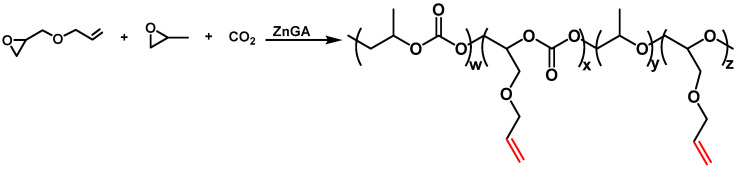
Terpolymerization of AGE, PO, and CO_2_ catalyzed by ZnGA.

The terpolymer synthesis is depicted in Scheme 1. CO_2_-based polycarbonate was synthesized from 17.43 g (0.3 mol) of propylene oxide (PO), 8.66 g (0.076 mol) of allyl glycidyl ether (AGE), and carbon dioxide at a pressure of 3.5 MPa. The polymerization process was conducted in a pre-dried 50 mL autoclave, in the presence of 0.5 g of a zinc glutarate catalyst. The process was carried out at 60 °C for 40 h under continuous magnetic stirring at 200 rpm. The resultant polycarbonate was dissolved in 30 mL of dichloromethane (DCM). Subsequently, it was precipitated by the addition of a mixture composed of ethanol (300 mL) and hydrochloric acid (15 mL) in a volumetric ratio of 20:1. This procedure was designed to extract unwanted cyclic carbonates, residual catalyst, and low molecular weight species from the product. After exhaustive washing, the polycarbonate was subjected to vacuum drying at 45 °C for a minimum of 12 h. The final product, the terpolymer PAGC, was obtained with a yield of 73%.

### 2.4. Synthesis of POSS-SH

According to the previously established literature [36,37,38], a synthesis was performed in a 1000 mL round-bottom flask, to which was added 500 mL of methanol (MeOH), 30 mL of (3-mercaptopropyl) trimethoxysilane (MPT), and 20 mL of concentrated hydrochloric acid. The reaction mixture was subjected to reflux at 90 °C for 36 h, under a continuous stream of nitrogen. Subsequent to the completion of the reaction, the resulting product was washed three times with cold MeOH to remove excess MPT. The concentrated solution was then redissolved in dichloromethane (DCM) and further purified with three sequential aqueous washes. The isolated compound was dried over anhydrous sodium sulfate, evaporated to concentrate, and finally subjected to desiccation, yielding the target silsesquioxane derivative, POSS-SH.

### 2.5. Preparation of PAGC-POSS x% Thin Films

**Scheme 2:**
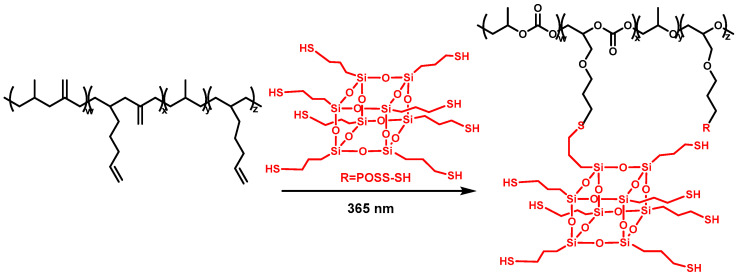
Preparation of the PAGC nanocomposites.

To synthesize PAGC-POSS x%, 1.6 g of PAGC and varying quantities of POSS-SH, including 0.045 g of the photo initiator DMPA, were dissolved in THF. The POSS-SH content was adjusted to 0.5%, 1%, 1.5%, and 2% relative to the quantity of PAGC. Subsequently, the solution was cast onto a tetrafluoro mold using a solution-casting method to form a film. The film was then cured under UV irradiation (ZF-1, light intensity of 6 mW cm^−2^) at room temperature for 6 h, followed by drying. The resulting films had thicknesses ranging from 0.4 to 0.5 mm. As illustrated in Scheme 2, the POSS-SH serves as a cross-linking agent that engages in a thiol-ene click reaction with the double bonds present in PAGC upon exposure to UV light.

## 3. Results and Discussion

### 3.1. Characterization of Carbon Dioxide-Based Polycarbonate Using PO and AGE as Raw Materials

**Figure 1:**
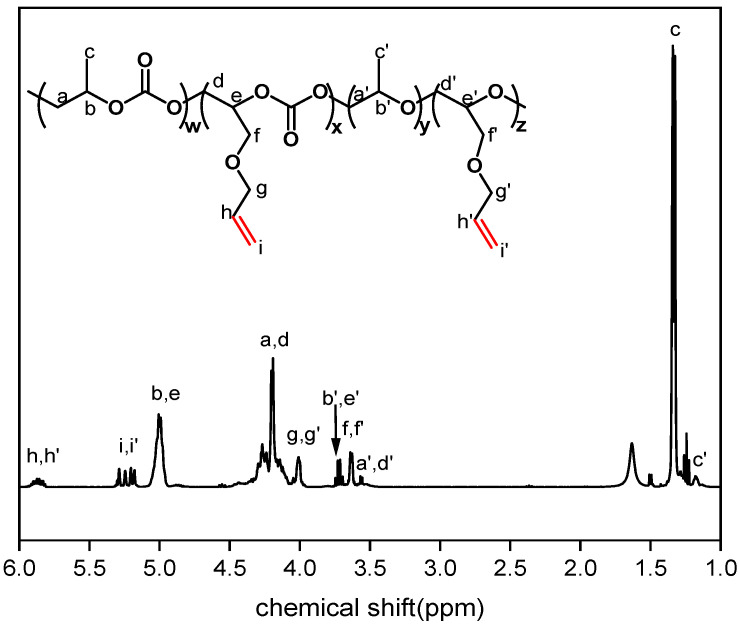
^1^H NMR spectra of PAGC obtained at feed ratio of PO to AGE as 8:2.

The chemical structure of the obtained terpolymers (PAGC) was characterized by ^1^H NMR spectroscopy [39], with the spectra represented in Figure 1. Chemical shifts observed at 5.8 ppm, and within the range of 5.1–5.3 ppm, were attributed to the allylic protons, corroborating the successful incorporation of the AGE monomer into the PPC backbone. Additionally, resonance peaks at 4.2 ppm and 5.0 ppm were identified, corresponding to the methylene and methine protons in the polycarbonate segment, respectively. The terpolymer was determined to have a molecular weight (*M*_n_) of 16,000, with a polydispersity index (*Đ*) of 1.99 (Figure S1).

### 3.2. Preparation and Characterization of the PAGC Nanocomposite Films

Previous research has demonstrated the synthesis of reactive polyhedral oligomeric silsesquioxane (POSS) using (3-mercaptopropyl) trimethoxysilane (MTS) as the precursor [37,38]. The successful synthesis of the compound POSS-SH is evidenced in Figure S2.

The degree of crosslinking within the hybrid materials can be modulated by varying the concentration of POSS-SH incorporated. The interaction between the sulfhydryl group in POSS-SH and the double bond in the polycarbonate led to the formation of crosslinks within the polymer matrix. This crosslinking reaction caused a structural transformation in the polycarbonate, transitioning it from a linear chain configuration to a more resilient three-dimensional arrangement.

**Figure 2:**
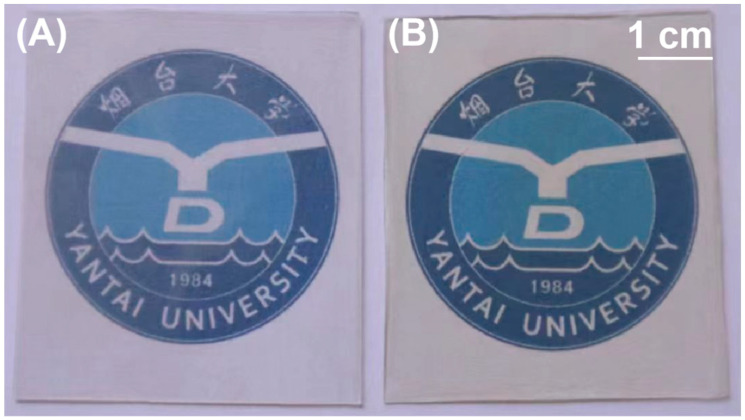
Visual comparison of PAGC (**A**) and PAGC-POSS 2% (**B**) films.

The transparency of membrane materials is crucial as it allows for effective light transmission, rendering the objects or surfaces behind them visible. This property holds significant importance across various domains. In packaging and protection sectors, transparent film materials find extensive utility in applications such as food packaging, protective coverings, and films. Essentially, transparent film materials serve as indispensable components in numerous industries, offering visually appealing and robust solutions for diverse applications. As depicted in Figure 2, image A depicts the pristine PAGC, while image B shows the composite films enriched with 2 wt% POSS-SH. These composites are distinguished by their uniformity and transparency, devoid of any discernible agglomeration, suggesting the absence of macroscopic phase separation. However, the PAGC-POSS x% nanocomposites exhibit a slight yellowish color when compared to the unmodified PAGC. This color change is attributed to the conversion of linear POSS-SH and PAGC structures into cross-linked networks by mercaptan-alkene click reaction. In contrast to the unmodified polycarbonate, the crosslinked polycarbonate exhibits a tighter molecular network, impeding light transmission and leading to a slight yellowing effect.

As illustrated in Figure 2, both the surfaces of crosslinked modified materials and pure polycarbonate exhibit a smooth appearance, with no noticeable agglomeration. Furthermore, it is worth noting that the surface of the film containing 2 wt% POSS appears smoother than that of pure PAGC. Maintaining transparency in composites, as suggested by Khanarian, typically requires the size of the dispersed phase to be smaller than 0.1 µm [40]. Therefore, it becomes evident that the size of the dispersed POSS phase within the PAGC matrix is indeed smaller than 0.1 µm. This size reduction is attributed to the thiol-ene click reaction, which forges a strong chemical bond between dispersed POSS-SH and PAGC, effectively enhancing the dispersion of POSS-SH within the PAGC.

**Figure 3:**
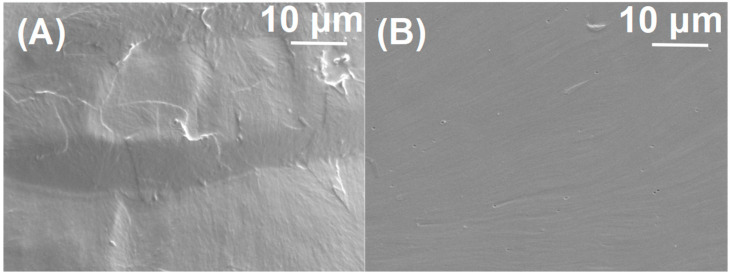
Analysis of fractured film surfaces (**A**) PAGC and (**B**) PAGC-POSS 2%.

The interfacial compatibility and homogeneity of POSS within the PAGC matrix were assessed by scrutinizing the cross-sectional morphology via scanning electron microscopy (SEM). Figure 3 presents the SEM micrographs of the fractured surfaces of both the PAGC-POSS 2% composite and the unmodified PAGC. Due to the relatively low glass transition temperature of PAGC-POSS x%, resulting in a soft film state at room temperature, achieving a complete cross-section becomes challenging. To prepare the sample for SEM, a freeze fracture technique is employed. By leveraging the low temperature of liquid nitrogen, a piece of membrane material is submerged in liquid nitrogen, rapidly hardening it. Subsequently, the membrane material is swiftly fractured from the middle to obtain a complete fracture surface of polycarbonate. This preparation method proves advantageous in maintaining phase morphology and accurately depicting the dispersed phase in the polymer matrix.

Elemental distribution maps enable visualization of the spatial arrangement of specific elements within a material, allowing for assessment of the uniformity of additive dispersion within a matrix. This uniformity is indicative of a successful synthesis and consistent distribution of the elements. In our study, the homogeneous dispersion of silicon (Si) and sulfur (S) elements within the polymer matrix is evidenced by the elemental distribution maps presented in Figures S3 and S4 (Supporting Information). These results highlight the effective incorporation of POSS-SH into the composite materials.

The microstructural morphology of a material is a critical determinant of its mechanical properties. Our experimental study focuses on the impact of polyhedral oligomeric silsesquioxane (POSS) on the phase morphology of polymer composites, facilitated by mercaptan-alkene reactions. The Atomic Force Microscope (AFM) was employed to investigate the composites and provided the viscoelasticity information from the phase images as shown Figure S5 in supporting information. Comprehensive scanning of various regions of the film was conducted, revealing distinct topographical features. The surface of the pristine polymer membrane is characterized as smooth and devoid of irregularities. Upon the addition of POSS-SH to PAGC, the ensuing mercaptan-alkene reaction gives rise to a phase structure with no apparent continuous phase separation. Notably, the emergence of protuberances in the middle regions, functioning as cross-linking junctions, is observed to augment the material properties, as delineated in Figure S5.

**Figure 4:**
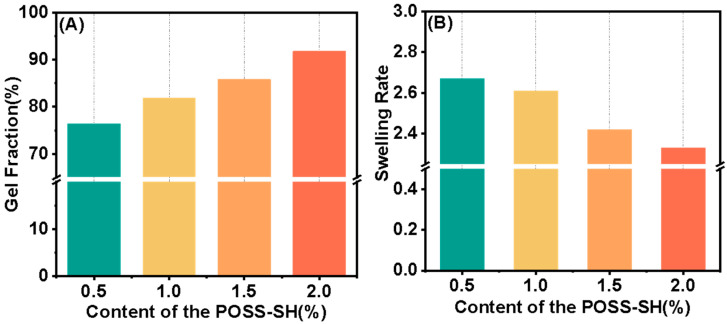
Gel fraction and swelling ratio of composites with varying POSS-SH Content. (**A**) Gel fraction of the composites as a function of POSS-SH content; (**B**) swelling ratio of the composites.

The successful execution of the thiol-ene click reaction between POSS-SH and PAGC is elucidated by the observed solubility changes and the quantification of gel content in tetrahydrofuran (THF). The composite’s gel content was ascertained by immersing the sample in THF at ambient temperature for 48 h. Subsequently, the sample was dried to a constant weight at 80 °C. Figure 4A shows the gel content of the samples post-reaction, which indicates that the degree of cross-linking within the polymer increases proportionally with the addition of POSS-SH. Specifically, a POSS-SH concentration of 0.5 wt% correlates with a gel content of 76.4%, whereas at 2 wt% POSS-SH, the gel content escalates to 91.8%. This pattern in the dissolution data supports the occurrence of the thiol-ene click reaction, which leads to enhanced cross-linking within the polymer matrix. As shown in Figure 4B, the swelling ratio of the PAGC-POSS x% composites diminishes from 2.67 to 2.33 when the POSS-SH content is increased from 0.5 wt% to 2 wt%. Consolidating these findings, it can be deduced that POSS-SH is well dispersed within the polymer matrix, thereby enhancing the uniform internal stability of the material. Furthermore, the reduction in swelling rate observed upon the incorporation of POSS-SH suggests an enhancement in the solvent resistance of the materials. This improvement can be attributed to the enhanced cross-linking structure, which leads to tighter binding among the internal molecules. As a result, the molecules become more intricately bound together, making them more resistant to the penetration and separation caused by solvents. This enhanced resistance to solvent action is crucial for applications where the material is exposed to various environmental conditions or chemical agents. Overall, the incorporation of POSS-SH contributes to the development of materials with improved durability and stability, expanding their potential utility in diverse fields.

### 3.3. Mechanical Properties of PAGC Composites

**Figure 5:**
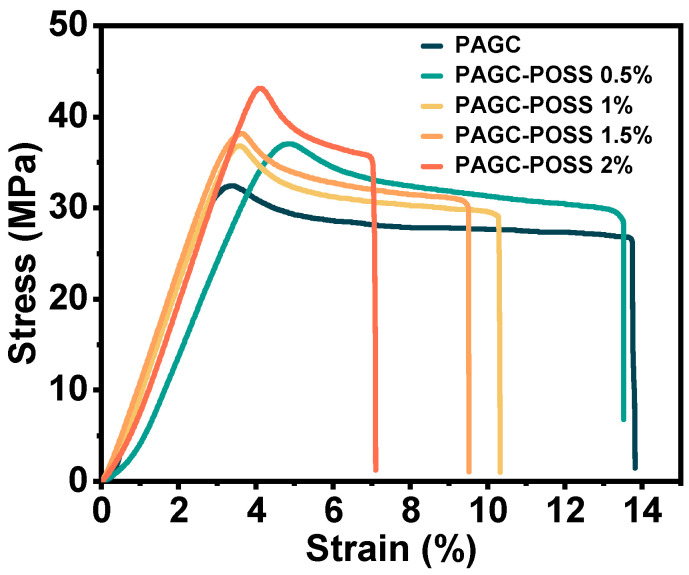
Stress-strain curves of PAGC and PAGC-POSS x% under constant temperature (20 °C).

To investigate the impact of POSS-SH addition on the mechanical properties of PAGC, membrane materials were prepared in dumbbell-shaped specimens, both before and after modification. Tensile tests were conducted at room temperature, utilizing a test speed of 5 mm/min. The stress-strain curves typical of elastomers, as depicted in Figure 5, were recorded. The mechanical properties of both pure PAGC films and those with varying POSS-SH contents were measured under standard conditions (20 °C, 20% relative humidity).

The mechanical properties of polycarbonate showed significant tunability with the incorporation of POSS-SH. The material strength notably increased from 32.5 MPa to 43 MPa with the addition of 2 wt% POSS-SH. Compared to the existing carbon dioxide-based polycarbonate (PPC) utilized in industrial sectors, the tensile strength of the carbon dioxide-based polycarbonate synthesized via copolymerization with carbon dioxide and propylene oxide (PO) typically falls within the range of 4.7 MPa to 21.5 MPa [41]. The mechanical strength of the carbon dioxide-based polycarbonate materials fabricated in this investigation exceeds that of the current industrial counterparts, highlighting their impressive mechanical properties. Furthermore, with the gradual incorporation of POSS-SH, the mechanical strength undergoes a systematic enhancement, showcasing a discernible trend of continuous improvement. Additionally, there is a notable decrease in the elongation at break, which reduces from 13.8% to 7.0% as the POSS-SH content increases, a change that can be attributed to the denser cross-linked network established between PAGC and POSS-SH via the click reaction. The increased incorporation of POSS-SH facilitates the formation of a more densely packed cross-linking network, fostering stronger interactions among molecules and tighter bonds between chains. As a result, this augmentation enhances the mechanical strength of the material while simultaneously reducing its elongation at break. Typically, an increase in material strength correlates with a decrease in elongation. Additionally, the elastomer tends to exhibit more plasticity with less elastic recovery, leading to reduced toughness. This behavior is a consequence of the higher cross-linking degree associated with increased POSS-SH content, which restricts the movement of polymer chains.

### 3.4. Thermomechanical Properties of PAGC Composites

**Figure 6:**
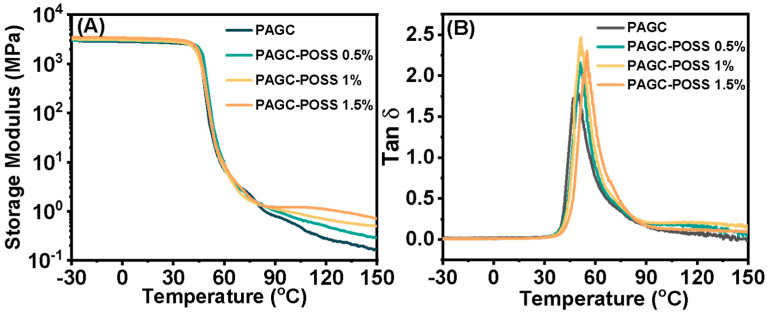
(**A**) The storage modulus curves PAGC and PAGC-POSS x%, (**B**) tan δ curves of PAGC and PAGC-POSS x%.

Figure 6 illustrates the dynamic thermomechanical analysis (DMA) spectra of the materials. Figure 6A demonstrates how the energy storage modulus of PAGC nanocomposites is affected by temperature. As the system transitions from a glassy to a rubbery state, a significant decrease in the storage modulus is observed, indicative of increased molecular chain mobility with rising temperature. Generally, a higher storage modulus denotes a material that is stiffer and more resistant to deformation. Conversely, a greater ability to store energy suggests improved elasticity. The introduction of POSS-SH into PAGC enhances its elastic recovery capabilities, underscoring the role of POSS-SH in the material’s modification and optimization.

Notably, the storage modulus within the plateau region for PAGC-POSS 1.5% surpasses that of PAGC-POSS 1%, a phenomenon attributable to the denser cross-linking network resulting from the thiol-ene click reaction between POSS and PAGC. With the incremental addition of POSS, both the cross-linking density and the energy storage modulus increase, signifying that POSS incorporation markedly boosts the storage modulus of PAGC across the entire temperature range examined.

In Figure 6B, the typical tan delta curve for PAGC nanocomposites indicates a glass transition temperature (*T*_g_) of around 50 °C. Beyond 100 °C, a pronounced fluctuation is observed, which correlates with the extent of polymer cross-linking. This suggests that as the quantity of POSS-SH incorporated increases, so does the material’s viscosity, reflecting the rise in cross-link density.

**Figure 7:**
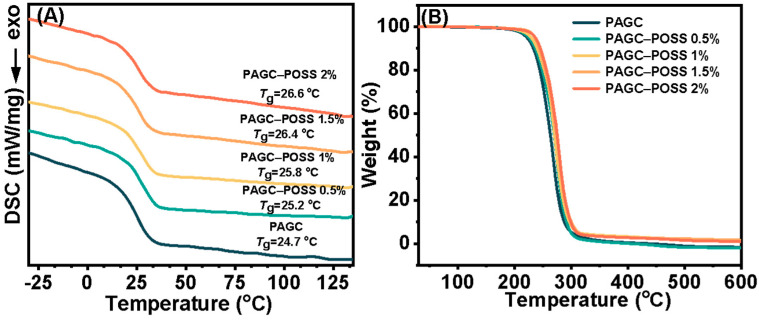
(**A**) DSC curves of PAGC and PAGC-POSS x%, (**B**) TG curves of PAGC and PAGC-POSS x%.

The glass transition temperature (*T*_g_) plays a pivotal role in determining the thermal stability and mechanical properties of polymers. The resulting ternary copolymer displays a distinct glass transition temperature (*T*_g_), which can be finely tuned by varying the amount of POSS-SH incorporated into the system. As shown in Figure 7A, the terpolymer maintains a single *T*_g_. Specifically, in our study, we observed a systematic trend in the *T*_g_ of the PAGC-POSS x% materials as the concentration of POSS-SH varied. Significantly, with an increase in the content of POSS-SH, there was a gradual rise in the *T*_g_ of the resultant materials. This observation suggests the impact of POSS-SH on the molecular structure and intermolecular interactions within the polymer matrix. The elevation in *T*_g_ indicates an enhancement in the thermal stability of the materials, attributable to the formation of a more interconnected and robust cross-linking network induced by POSS-SH. The presence of POSS-SH molecules facilitates the establishment of additional cross-links between polymer chains, thereby restricting molecular mobility and impeding segmental motion at elevated temperatures. Consequently, this phenomenon contributes to the improved thermal performance of the PAGC-POSS x% composites. Enhanced cross-linking confers greater structural rigidity and improved thermal stability to the polymer. The observed discrepancies in *T*_g_ values acquired from DMA and DSC are attributable to the differing measurement principles and algorithms used by these techniques. DSC estimates *T*_g_ based on specific heat changes, with accuracy potentially diminished by factors such as dilution, crystallization, and cross-linking. Conversely, DMA measures significant changes in viscoelastic properties, reflecting the progression of molecular motion from localized atomic vibrations to larger-scale chain movements.

Thermogravimetric analysis can be used to analyze and study the thermal stability of materials. Figure 7B presents the thermogravimetric analysis (TGA) spectra, comparing pure poly (allyl glycidyl carbonate) (PAGC) with PAGC crosslinked with 1.5% polyhedral oligomeric silsesquioxane octamercaptopropyl (POSS-SH) nanocomposites. The data indicate that the integration of POSS-SH into the PAGC matrix results in an increased rate of residual carbon, indicative of an enhanced resistance to thermal degradation. This improvement in thermal stability is attributable to the formation of a denser crosslinked network between the PAGC chains and the POSS-SH entities, thereby yielding a PAGC-POSS composite with relatively high thermal stability.

## 4. Conclusions

By utilizing zinc glutarate as a catalyst, we successfully facilitated the ternary copolymerization of propylene oxide (PO), allyl glycidyl ether (AGE), and carbon dioxide (CO_2_), resulting in a CO_2_-based polycarbonate (PAGC) with double-bond active sites in the side chain. The subsequent incorporation of thiol-functionalized polyhedral oligomeric silsesquioxane octamercaptopropyl (POSS-SH) into the PAGC via UV initiation has resulted in the formation of PAGC-POSS x% polycarbonates. These polycarbonates are distinguished by their exceptional optical clarity, superior mechanical strength, and enhanced thermal properties. The formation of a micro-crosslinked network through a thiol-ene click chemistry reaction has notably augmented the tensile strength of the resulting polycarbonate to 42 MPa, marking a significant enhancement over conventional CO_2_-based polycarbonates. Furthermore, the cross-linking structure enhances the resistance to organic solvents, resulting in materials with improved properties compared to traditional polycarbonates. This research broadens the application and theoretical foundation of PAGC in various plastic materials.

## Data Availability

Data are contained within the article.

## Supplementary Materials

The following supporting information can be downloaded at: https://www.mdpi.com/article/10.3390/polym16070983/s1, Figure S1: Molecular weight and molecular weight distribution of PAGC; Figure S2: (A) FTIR of POSS-SH, (B)^13^C NMR spectrum of POSS-SH in CDCl3, (C) ^1^H NMR of POSS-SH in CDCl3, (D) ^29^Si NMR of POSS-SH in CDCl3; Figure S3: EDS images of element S: (A) PAGC-POSS 0.5%, (B) PAGC-POSS 1%, (C) PAGC-POSS 1.5%, (D) PAGC-POSS 2%; Figure S4: EDS images of element Si: (A) PAGC-POSS 0.5%, (B) PAGC-POSS 1%, (C) PAGC-POSS 1.5%, (D) PAGC-POSS 2%; Figure S5 AFM phase images: (A) PAGC, (B) PAGC-POSS 2%.
