# Supplementary material for: Organic–Inorganic Hybrid Materials: Tailoring Carbon Dioxide-Based Polycarbonate with POSS-SH Crosslinking
Source: Polymers (Basel). 2024 Apr 4;16(7):983. doi: 10.3390/polym16070983 (PMC11013663; doi:10.3390/polym16070983)
Supplement: Supplementary file 1 [file polymers-16-00983-s001.zip › polymers-2930528-SI.pdf]

Supporting Information

Organic-Inorganic Hybrid Materials: Tailoring Carbon Dioxide-Based Polycarbonate with POSS-SH Crosslinking

Yue Li [a], Jianyu Liu [a,b], Rui Qu [a,b], Hongyi Suo [a,b], Miao Sun [a,b] *, Yusheng Qin [a,b] *
[a] College of Chemistry and Chemical Engineering, Yantai University, Yantai 264005, Shandong, China.
[b] Institute of Materials, Yantai University, Yantai 264005, Shandong, China.

Swelling Tests. The assessment of gel content and swelling ratio involved conducting equilibrium swelling experiments in tetrahydrofuran (THF) based on the Flory-Rehner equation. The samples were subjected to equilibrium swelling experiments by immersing them in THF at room temperature for 72 hours, with the solvent being replaced with fresh THF every 24 hours. After the swelling process, the solvent was promptly removed from the sample surface using filter paper, and the samples were immediately weighed. Subsequently, they were dried in a vacuum oven at 80 °C until a constant weight was achieved. Three specimens were measured for each sample. The swelling ratio was calculated as $(m_1-m_2)/m_2$, and the sol fraction was determined as $(m_0-m_2)/m_0$, where m_0 represents the sample mass before swelling, and m_1 and m_2 are the weights of the swollen and deswollen samples, respectively.

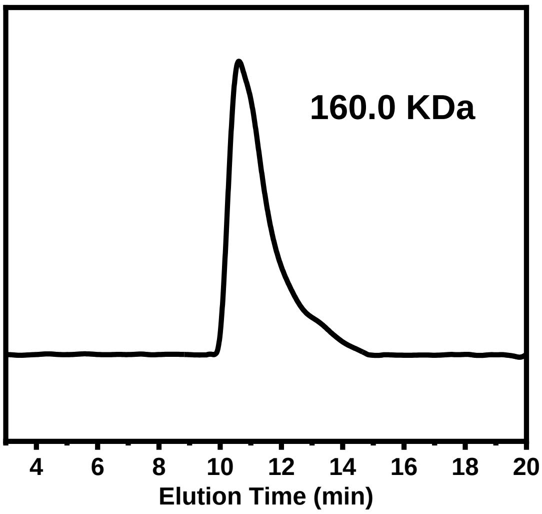

Figure S1. GPC curves of the obtained PAGC.

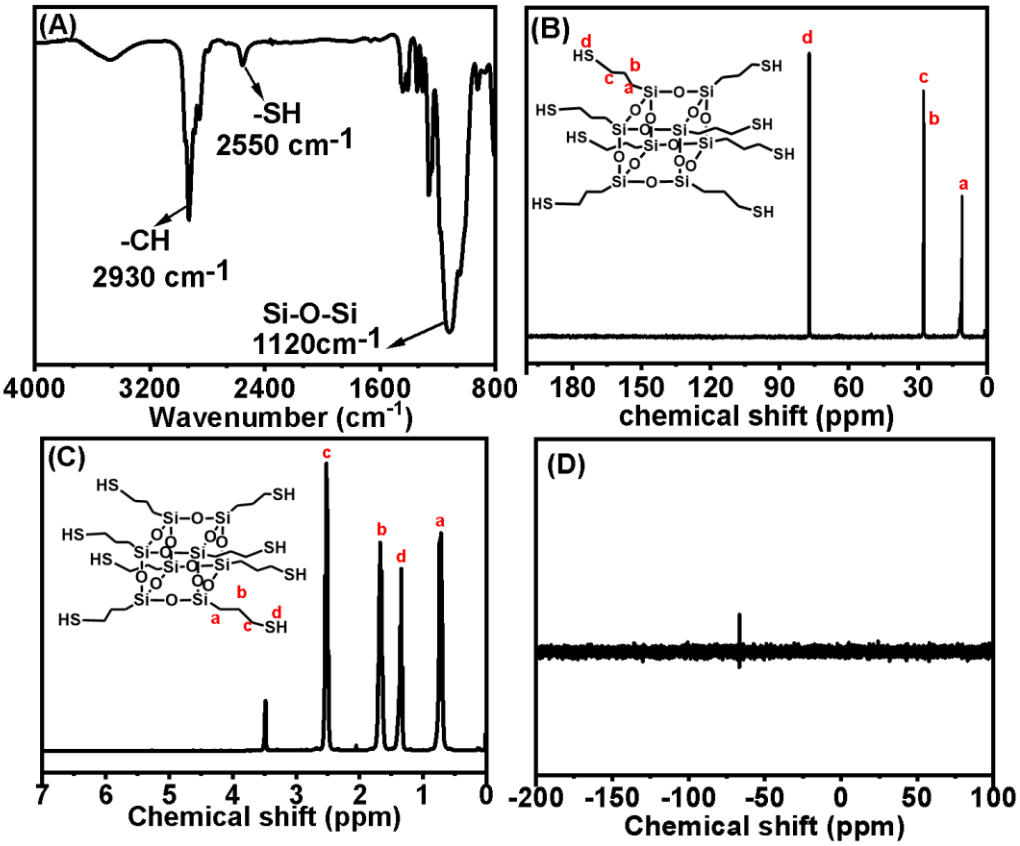

Figure S2. (A) FTIR of POSS-SH, (B)^{13}C NMR spectrum of POSS-SH in $CDCl_3$, (C) ^{1}H NMR of POSS-SH in $CDCl_3$, (D) ^{29}Si NMR of POSS-SH in $CDCl_3$.

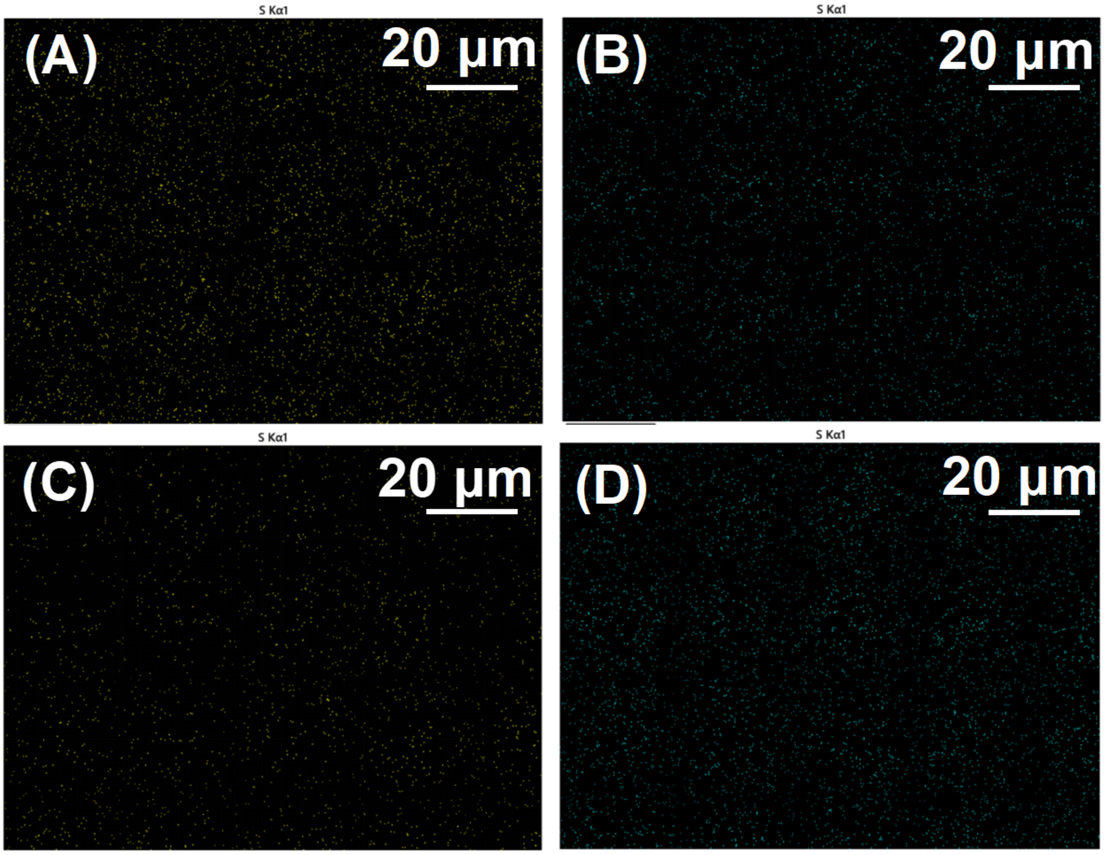

Figure S3. EDS images of element S: (A) PAGC-POSS 0.5%, (B) PAGC-POSS 1%, (C) PAGC-POSS 1.5%, (D) PAGC-POSS 2%.

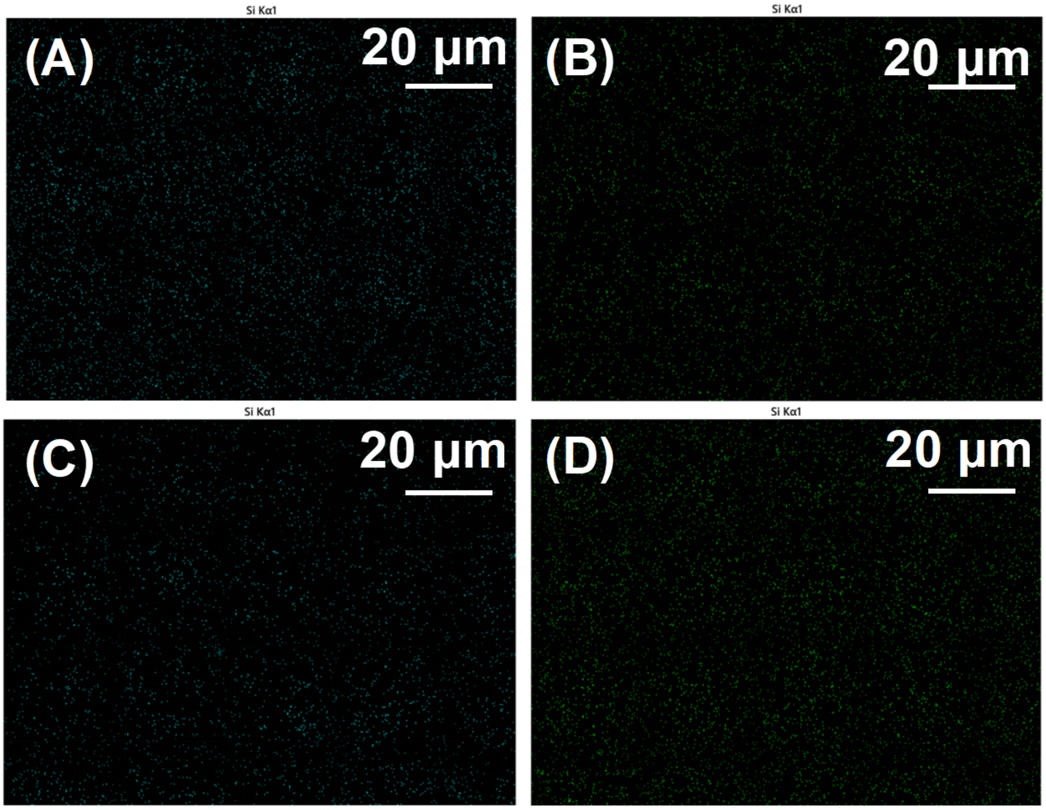

Figure S4. EDS images of element Si: (A) PAGC-POSS 0.5%, (B) PAGC-POSS 1%, (C) PAGC-POSS 1.5%, (D) PAGC-POSS 2%.

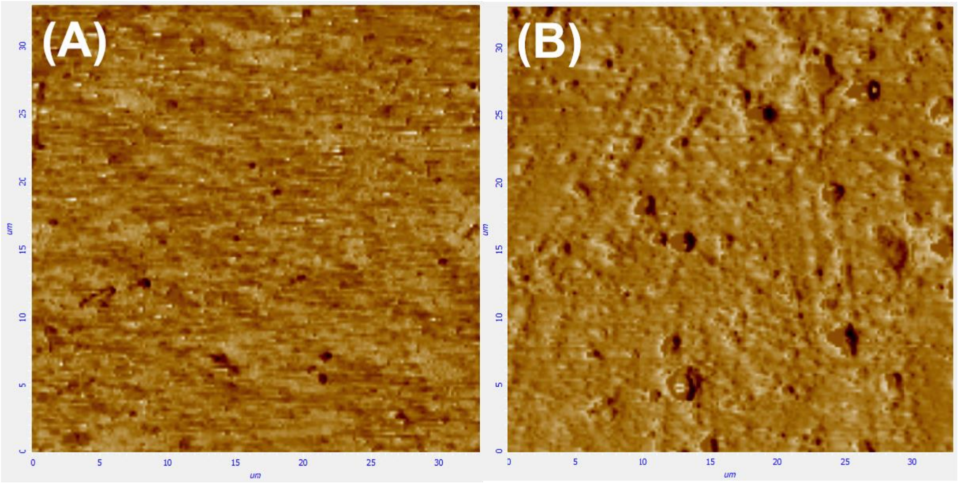

Figure S5. AFM phase images: (A) PAGC, (B) PAGC-POSS 2%.
